# Comparison of Body Composition Methods for Estimating Body Fat Percentage in Lower Limb Prosthesis Users

**DOI:** 10.33137/cpoj.v6i1.41605

**Published:** 2023-11-10

**Authors:** JD Smith, G Guerra, TB Symons, EH Kwon, EJ Yoon

**Affiliations:** 1 Department of Counseling, Health and Kinesiology, College of Education and Human Development, Texas A&M University-San Antonio, San Antonio, Texas, USA.; 2 Department of Exercise and Sport Science, St. Mary's University, San Antonio, Texas, USA.; 3 Laboratory of Animal Physiology and Medicine, Department of Biology Education, Korea National University of Education, Chungbuk, Republic of Korea.

**Keywords:** Body Composition, Prosthesis, DXA, ADP, Air Displacement Plethysmography, Dual-Energy X-Ray Absorptiometry, Body Fat, Amputation

## Abstract

**BACKGROUND:**

There is a dearth of literature evaluating the accuracy of Air Displacement Plethysmography (ADP) compared to Dual-energy X-ray Absorptiometry (DXA) for assessing body composition in individuals with lower limb amputations. Validity of ADP in persons with lower limb amputations must be established.

**OBJECTIVE:**

The objective of this study was to compare body composition in persons with lower limb amputations using the BOD POD^®^ and DXA.

**METHODOLOGY:**

Body composition was performed on eleven lower limb prosthesis users (age 53.2±14.3 years, weight 81.9±22.3kg) using ADP and DXA with and without prosthesis.

**FINDINGS:**

Repeated measures ANOVA indicated no significant difference in body composition among and between trials, F(3,8)= 3.36, p= 0.075. There were no significant differences in Body Fat (BF) percentage with and without prostheses on the BOD POD (28.5±15.7% and 33.7±12.1%, respectively) nor the DXA (32.9±10.6% and 32.0±9.9%, respectively). Association between the BOD POD and DXA were greatest when prostheses were not worn compared to when they were worn. Bland-Altman plots indicate agreement between BOD POD^®^ and DXA was greatest while wearing the prosthesis.

**CONCLUSION:**

This study is a first to compare total body fat percent between the BOD POD^®^ and DXA in lower limb prosthesis users. BOD POD^®^ report valid indices of BF%. Future work will utilize the BOD POD^®^ in intervention studies for monitoring body composition changes across the continuum of rehabilitation.

## INTRODUCTION

In the United States over forty percent of individuals are considered obese.^1^ Persons with a higher percentage of body fat are at greater risk of negative cardiometabolic health.^2,3^ Likewise, higher body mass index (BMI) may increase susceptibility to coronary heart disease and type 2 diabetes,^4–6^ and increase medical expenses.^7,8^ There is also evidence that individuals who are obese may suffer from lowered wages and education.^9^ Prevalence of obesity may be higher in persons with lower limb amputation (LLA) than in able-bodied persons,^10–12^ with higher BMI typically seen at higher amputation levels (i.e. trans-femoral).^13,14^ For those having received dysvascular-related amputations, loss of adequate blood flow to the peripheries, negatively alters limb musculature and fat mass.^15,16^ Most people receive amputation as a result of dysvascular disease and it is these individuals that are often overweight with a BMI ≥ 25 kg/m2 or even obese with a BMI ≥ 30 kg/m2.^17,18^ Although BMI is simple to calculate, body fat percentage (BF%) provides a better indicator of health risk.^19^ A closer analysis of limb composition in people with amputation has found greater muscle atrophy and fat mass (FM) in the amputated limb.^20^ Obesity is associated with dysvascular disease, which accounts for a majority of lower extremity amputations in the United States, thus, valid body composition assessment is essential.

A variety of commercial and research grade tools exist for measuring body composition.^21^ Bioelectrical Impedance Analysis (BIA) is inexpensive, portable and non-invasive.^22^ Despite BIA being validated and used in research,^23–25^ error of 9.79% has been seen when compared to Dual-energy X-ray Absorptiometry (DXA).^26^ The DXA offers precise estimates of body Fat Free Mass (FFM),^27^ but its high cost and radiation exposure makes routine use prohibitive. Air Displacement Plethysmography (ADP) is not as costly as DXA and requires limited training to administer.^25^

There is a dearth of literature comparing total BF% from ADP to DXA in persons with lower limb amputations. Those that have explored validation have found that ADP can overestimate BF% in thinner individuals and underestimate BF% in heavier persons.^28^ For body composition assessment to become a routine outcome measurement, time and resources must be considered.^29^ ADP may be widely utilized, however, its validity in persons with lower limb amputations must still be established. The purpose of this study was to evaluate body composition as measured by ADP when compared to DXA in persons with lower limb amputations.

Since there are a lack of studies that compare percent body fat of those with lower limb amputations using ADP and DXA, ^1^) it was hypothesized there would be no significant difference in percent body fat between ADP and DXA and ^2^) it was also hypothesized there would be no significant difference in percent body fat when wearing and not wearing prosthesis.

## METHODOLOGY

### Participants

This study was approved by the Texas A&M University San Antonio Institutional Review Board (Log#2020-69) and all participants signed an informed consent form before study commencement. Eleven persons with limb amputations participated in this study (**Table 1**) and were recruited in San Antonio, TX through local support groups and clinics. The selection criteria included any person with a lower limb amputation who could ambulate on a prosthesis with or without an assisted device, and excluded those who might be pregnant. This sample size reflects that of other studies utilizing DXA as a measure of body composition.^30–33^ All participants were asked to not eat a heavy meal four hours prior to testing, abstain from exercise, and maintain appropriate hydration prior to data collection. Height was measured while wearing prostheses and no shoes to the nearest 0.1 cm using a stadiometer (Seca^®^ 213, Hamburg, Germany). Body mass was measured to the nearest 0.1kg using a digital scale (BOD POD^®^, COSMED, USA Inc., Concord, CA, USA). Body mass index was determined by weight divided by stature squared (kg/m2). Assessments were performed by trained professionals with university employment and all measurements were carried out according to manufacturer instructions.

**Table 1:** Participant characteristics mean±SD.

	N=11	Amputation Level	
Age (yrs)	53.2±14.3	Below Knee	8
Height (cm)	170.7±9.2	Above Knee	1
Weight (kg, with prostheses)	84.7±21.8	Bilateral Below Knee	1
Weight (kg, without prostheses)	81.9±22.3	Other*	1
BMI (with prostheses)	28.9±6.1		
BMI (without prostheses)	27.8±6.2		

* Note: Other is right hip disarticulate and left below knee.

### Measurements

Participants reported on two separate days and prior to measurement, were asked to empty their bladder. The ADP body volume measurement was conducted using a BOD POD^®^ (COSMED USA Inc., Concord, CA, USA). A warmup, calibration and weight scale calibration were performed before testing according to the manufacturer guidelines. Participants removed all jewelry and wore tight-fitting garments (swimsuits, yoga pants, etc.) and swim cap to reduce air trapped in clothing and hair. Body mass was measured using the BOD POD^®^ scale and volume determined twice. Participants sat quietly and breathed normally while seated in the BOD POD^®^. The measurement procedure was repeated once again after doffing the prosthesis. The COSMED software's Siri equation was chosen for measurements of BF%.^34,35^ Whole body DXA measurement occurred immediately after the BOD POD^®^ measurement using a Lunar Prodigy (GE Medical Systems, Chicago, IL). Before measurement, the DXA was calibrated according to manufacturer guidelines. Participants wore the same minimal tight-fitting clothing worn during BOD POD^®^ measurements and the same investigator performed and analyzed each scan. Participants were asked to lay supine with limbs oriented by their sides, once with the prosthesis and once again without wearing prosthesis. This procedure took 20–30 minutes and once complete, participants were scheduled for their second session, which took place approximately one week later.

### Statistical analysis

Analysis was performed using SPSS version 27 (IBM Corp, Armonk, NY). A repeated measures ANOVA was performed to explore differences in body composition among and between the trials, a method commonly used to make these types of comparisons (CITE).^36–40^ Bland and Altman plots were created to assess the agreement between the two body composition methods. Pearson's Product Moment correlations were performed to determine relationships between the two methods, with 0.20–0.39 being weak, 0.40–0.59 being moderate, 0.60–0.79 being strong, and >0.80 being very strong.^41^ Cronbach's Alpha was used as a measure of internal consistency with 0.90 or greater considered high agreement; 0.80 to 0.89, moderate agreement; and 0.79 or lower, low agreement^42^ Alpha of 0.05 was used for all statistical testing.

## RESULTS

**Figure 1:**
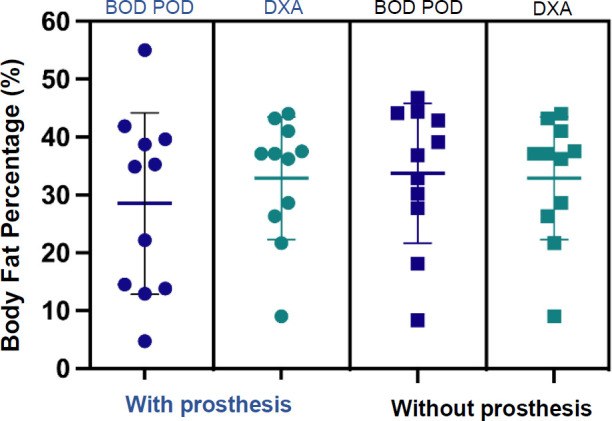
There were no significant differences in percent body fat between BOD POD^®^ and DXA when wearing and not wearing prostheses, p > 0.05. Note: Individual data points are participant data points for BOD POD^®^ and DXA with and without prosthesis.

**Figure 2:**
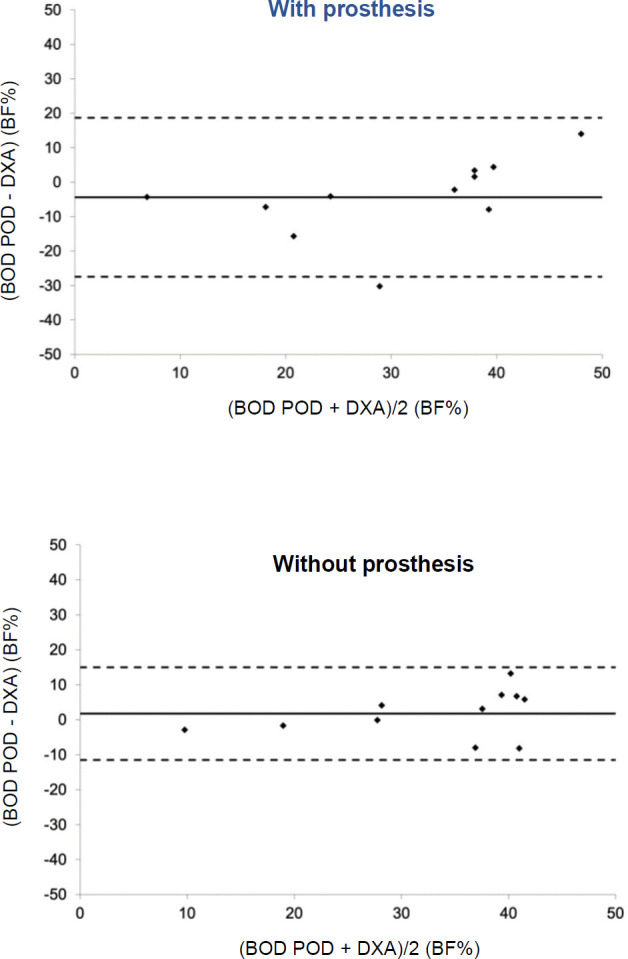
Tighter limits of agreement exist between the BOD POD^®^ and DXA when not wearing prostheses. Note: Solid line represents mean difference and dashed lines represent 95% limits of agreement.

**Table 1** provides participant demographics and prosthesis type. Results of repeated measures ANOVA on body composition among and between trials were not significant, F(3,8)= 3.36, p= 0.075. While there was a trend for BF% measured by the BOD POD to be lowest with the prosthesis (28.5±15.7%) and highest without it (33.7±12.1%), measurements by the DXA for both with (32.9±10.6%) and without (32.0±9.9%) fell between those parameters (**Figure 1**), none of which were significant. It is worthy to note, however, that the effect size for BOD POD^®^ (Cohen's d = 0.71) is moderate compared to DXA (Cohen's d = 0.32), which is weak.^43^ Bland-Altman analysis indicated mean difference and 95% limits of agreement (LOA) between the BOD POD^®^ and DXA was greatest when wearing prostheses (mdiff = −4.38, 95% LOA = −27.4 −18.7) compared to not wearing the prostheses (mdiff = 1.73, 95% LOA = −11.5 −14.9), **Figure 2**.

**Figure 3:**
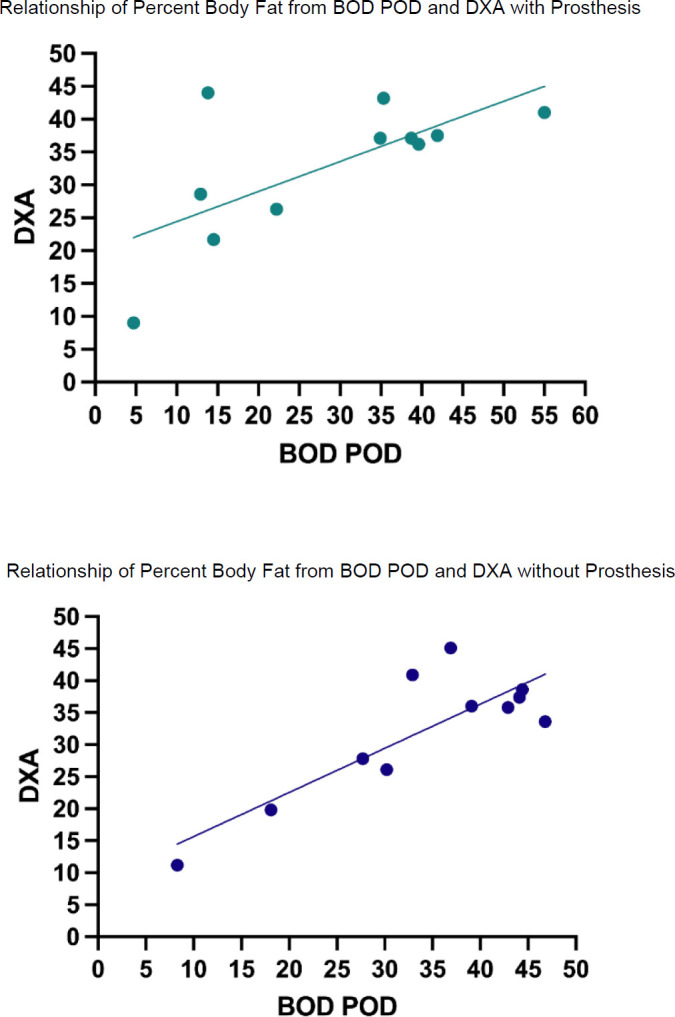
Relationships of percent body fat from BOD POD^®^ and DXA with and without prosthesis. Note: There was a stronger association in percent body fat between BOD POD^®^ and DXA when not wearing prostheses (r(9) = 0.87, p = 0.001) compared to wearing it (r(9) = 0.68, p = 0.02) during measurements. The (9) represents the degrees of freedom.

**Table 2** provides a correlation matrix depicting the relationships of the measures, all of which were significant at 0.05. The highest Pearson correlations between the BOD POD^®^ and DXA were observed between devices when prostheses were not worn compared to when they were worn (**Figure 3**). Similarly, internal consistency was greatest between the two instruments while not wearing prostheses (Cronbach's Alpha = 0.901) compared to wearing them (Cronbach's Alpha = 0.772).

**Table 2:** Correlations of percent body fat from BOD POD^®^ and DXA with and without prostheses.

	BOD POD^®^ With	BOD POD^®^ Without	DXA With
BOD POD ^®^ Without	0.89^*^		
DXA With	0.68*	0.87*	
DXA Without	0.63*	0.84*	0.97*

Note:

* *p* < 0.05

## DISCUSSION

In this study we compared BOD POD^®^ derived body fat percent estimates to DXA estimates in persons with lower limb prosthesis. We found that BOD POD^®^ estimates were not significantly different to DXA estimates whether prosthesis was worn or not. The BOD POD^®^ underestimated body fat percentage compared to DXA when wearing the prosthesis by 13.1% (difference of 4.3%) and overestimated without the prosthesis 5% (difference of 1.7%), respectively.

Some studies have observed divergences of –3.0% to 1.7%, ^44^ while others have observed 1.6% overestimates of BF% for BOD POD^®^ over DXA %BF. Still, the novelty of our study is our finding of similar measures of BOD POD^®^ and DXA in lower limb prosthesis users. The instruments compared in our study have been compared in the general population, ^45^ persons with diabetes and obesity, ^46^ and even wheelchair sport users.^47^ The differences observed between the BOD POD^®^ and DXA may be a result of the BOD POD^®^ using a Siri 2-compartment model of fat and lean mass to calculate body fat percentage. The Siri densiometry formula estimates body fat from body density. This formula has been shown to overestimate BF% is non-obese active individuals,^48^ as well as individuals that are obese.^49^ As the body contains other tissues such as water, connective, and bone, distinguishing bone density is possible with DXA, which may help reduce variances. However, it is unclear at this time if specific formulas to assess body fat percentages in persons with lower limb amputations are required. The BOD POD^®^ is a suitable alternative to DXA as it places low burden on the prosthesis user, less expensive, simple to perform, and no need for radiation safety measures.

Greater agreement between the two measures as evidenced by tighter limits in the Bland-Altman plots suggest doffing the prostheses when assessing body composition. This is consistent with measures of internal consistency as calculated by Cronbach's Alpha. Furthermore, while the relationship between BF% when wearing the prostheses was moderately strong, doffing the prostheses produced the strongest relationship between the two measures. Given this information, and since there was a ~2% difference in BF% between BOD POD^®^ and DXA, it may be more clinically appropriate to assess BF% without prostheses when using either of these instruments.

The BOD POD^®^ itself may be subject to measurement error related to isothermal air used to determine raw body volume (BVr). An incorrect raw body volume might underestimate body volume (BV) which in turn may overestimate body density (BD) leading to an underestimated and imprecise BF%. Isothermal air trapped in clothing produces error,^50^ and may underestimate BF% in normal and overweight persons when loose fit clothing is compared with swimsuits or no clothing.^51^ As such, the present study suggests removal of the prosthesis during body composition assessment.

Although we did not investigate the ability of the BOD POD^®^ to track body composition changes, this is a popular method of in weight loss research. The BOD POD^®^ has been validated with DXA in several weight loss intervention studies with mixed results. In one study overweight participants were randomized to either a self-help group or weight loss program.^52^ At both pre and post measurements, BF% were lower in DXA than BOD POD^®^.^52^ However, two other studies found BOD POD^®^ to underestimate BF% and overestimate fat free mass (FFM) compared to DXA.^53,54^ This trend was evident in the current study, where BF% from the BOD POD^®^ was lower than from the DXA while wearing prostheses.

The results from this study suggest, that the BOD POD^®^ is a suitable option over the more resource demanding DXA. Dual-energy X-ray absorptiometry might have an inability to discern changes in components of fat free mass components as this method assumes adequate hydration of FFM.^55^ However, we selected DXA as our criterion as opposed to hydrostatic weight as DXA considers bone density and is more precise in measurement than the BOD POD^®^.^56^ As such, we believe differences seen between BOD POD^®^ and DXA are because of limitations of the BOD POD^®^ and not necessarily our sample of participants. Furthermore, while some participants had to be assisted on the body weight scale without prostheses prior to BOD POD^®^ measurements, entering the chamber itself was safe and not cumbersome. Some participants preferred to transfer themselves from a wheelchair to the chamber while others preferred to seat themselves in the chamber while wearing the prosthesis and then remove it just prior to measurement.

### Limitations

Our study is not without limitations. Our study was limited by a smaller sample with limited number of persons with transfemoral and bilateral amputations. Subject selection may have influenced the results because of the unique anthropometry of participants with amputations. Moreover, our group of participants had a BMI of 28.1 corresponding to an overweight classification (25.0 to 29.9 kg/m2). This may limit generalizability of our findings for those with lower limb amputations in other BMI ranges. Still, others have reported BMI of 31.7 in people with dysvascular amputation,^17^ and a study of 16,259 individuals with non-traumatic amputation found 30.4% to be non-obese, 18.2% obesity class 1 (BMI= 30–34.9), 17.3% obesity class 2 (BMI= 35–39.9), and finally 34.1% with obesity class 3 (BMI≥40).^57^

Although the BOD POD^®^ is a valid and reliable technique when estimating total BF% compared to the DXA, there are distinct advantages and disadvantages of either system. They are both simple to administer by practitioners and easy to perform by patients. The BOD POD^®^ is a less costly technique with no exposure to the very low radiation produced by the DXA. However, the DXA provides total values of FM, FFM and bone bass which differs from the BOD POD^®^ which measures the relative concentrations in estimating tissue content. Regardless, the BOD POD^®^ is widely acceptable for body composition testing for a range of patient populations. Our study suggests that it is also acceptable for measurement of body composition in lower limb prosthesis users.

## CONCLUSION

The rise of obesity around the world has ushered in a need for interventions as well as way of measuring body composition. Assessing body weight and BMI changes alone is misleading and cannot measure changes in lean mass or fat mass. This study is a first to assess differences of the BOD POD^®^ to DXA in lower limb prosthesis users. The results of this study indicate that when considering measuring BF% with BOD POD^®^ using DXA as a criterion, BOD POD^®^ reports less difference and greater agreement of BF% when doffing the prostheses. Future work will utilize the BOD POD^®^ in intervention studies for monitoring body composition changes across the continuum of rehabilitation.

## DECLARATION OF CONFLICTING INTERESTS

The authors declare no financial and personal relationships with organizations or individuals that might have influenced their research.

## AUTHORS CONTRIBUTION

- **John D. Smith:** original drafting, writing, data collection, statistical analysis.
- **Gary Guerra:** conceptualization, writing, data collection, statistical analysis.
- **T. Brock Symons:** writing, data collection, proofing.
- **Eun Hye Kwon:** writing, data collection, proofing.
- **Eun Jung Yoon:** writing, data collection, proofing.

## SOURCES OF SUPPORT

This research was supported by the COEHD Faculty Grant Program.

## ETHICAL APPROVAL

This study was approved by the Texas A&M University San Antonio Institutional Review Board (Log#2020-69) and all participants signed an informed consent form before study commencement.

## References

[1] Hales CM, Carroll MD, Fryar CD, Ogden CL. Prevalence of Obesity and Severe Obesity Among Adults: United States, 2017–2018. NCHS Data Brief. 2020; 360:1–8. PMID: 32487284 32487284

[2] Ito H, Nakasuga K, Ohshima A, Sakai Y, Maruyama T, Kaji Y, et al. Excess accumulation of body fat is related to dyslipidemia in normal-weight subjects. Int J Obes Relat Metab Disord. 2004;28(2):242–7. DOI:10.1038/sj.ijo.0802528 14610531

[3] Kim JY, Han SH, Yang BM. Implication of high-body-fat percentage on cardiometabolic risk in middle-aged, healthy, normal-weight adults. Obesity (Silver Spring). 2013;21(8):1571–7. DOI: 10.1002/oby.20020. PMID: 23404833 23404833

[4] Manson JE, Willett WC, Stampfer MJ, Colditz GA, Hunter DJ, Hankinson SE, et al. Body weight and mortality among women. N Engl J Med. 1995;333(11):677–85. DOI: 10.1056/NEJM199509143331101 7637744

[5] Haslam DW, James WPT. Obesity. Lancet. 2005;366(9492): 1197–209. DOI: 10.1016/S0140-6736(05)67483-1 16198769

[6] Kopelman PG. Obesity as a medical problem. Nature. 2000;404(6778):635–43. DOI: 10.1038/35007508 10766250

[7] Tsai AG, Williamson DF, Glick HA. Direct medical cost of overweight and obesity in the USA: a quantitative systematic review. Obes Rev. 2011;12(1):50–61. DOI: 10.1111/j.1467-789X.2009.00708.x 20059703 PMC2891924

[8] Cawley J, Biener A, Meyerhoefer C, Ding Y, Zvenyach T, Smolarz BG, et al. Direct medical costs of obesity in the United States and the most populous states. J Manag care Spec Pharm. 2021;27(3):354–66. DOI: 10.18553/jmcp.2021.20410 33470881 PMC10394178

[9] Apovian CM. Obesity: definition, comorbidities, causes, and burden. Am J Manag Care. 2016;22(7 Suppl):s176–85. PMID: 27356115 27356115

[10] Mollee TS, Dijkstra PU, Dekker R, Geertzen JHB. The association between body mass index and skin problems in persons with a lower limb amputation: an observational study. BMC Musculoskelet Disord. 2021;22(1):769. DOI: 10.1186/s12891-021-04646-2 34503484 PMC8428047

[11] Shahriar SH, Masumi M, Edjtehadi F, Soroush MR, Soveid M, Mousavi B. Cardiovascular risk factors among males with war-related bilateral lower limb amputation. Mil Med. 2009;174(10):1108–12. DOI: 10.7205/milmed-d-00-0109 19891226

[12] George BG, Pruziner AL, Andrews AM. Circumference method estimates percent body fat in male US service members with lower limb loss. J Acad Nutr Diet. 2021;121(7):1327–34. DOI: 10.1016/j.jand.2021.02.009 33744234

[13] Kurdibaylo SF. Obesity and metabolic disorders in adults with lower limb amputation. J Rehabil Res Dev. 1996;33(4):387–94. 8895133

[14] Tzamaloukas AH, Patron A, Malhotra D. Body mass index in amputees. JPEN J Parenter Enteral Nutr. 1994;18(4):355–8. DOI: 10.1177/014860719401800414 7933444

[15] McDermott MM, Hoff F, Ferrucci L, Pearce WH, Guralnik JM, Tian L, et al. Lower extremity ischemia, calf skeletal muscle characteristics, and functional impairment in peripheral arterial disease. J Am Geriatr Soc. 2007;55(3):400–6. DOI: 10.1111/j.1532-5415.2007.01092.x 17341243 PMC2645649

[16] Ma KF, Levolger S, Vedder IR, El Moumni M, de Vries J-PPM, Bokkers RPH, et al. The impact of lower extremity skeletal muscle atrophy and myosteatosis on revascularization outcomes in patients with peripheral arterial disease. J Clin Med. 2021;10(17):3963. DOI: 10.3390/jcm10173963 34501412 PMC8432022

[17] Rosenberg DE, Turner AP, Littman AJ, Williams RM, Norvell DC, Hakimi KM, et al. Body mass index patterns following dysvascular lower extremity amputation. Disabil Rehabil. 2013; 25;35(15):1269–75. DOI: 10.3109/09638288.2012.726690 23094934 PMC7546544

[18] Fard B, Dijkstra PU, Voesten HGJM, Geertzen JHB. Mortality, reamputation, and preoperative comorbidities in patients undergoing dysvascular lower limb amputation. Ann Vasc Surg. 2020;64:228–38. DOI: 10.1016/j.avsg.2019.09.010 31629839

[19] Zeng Q, Dong S-Y, Sun X-N, Xie J, Cui Y. Percent body fat is a better predictor of cardiovascular risk factors than body mass index. Brazilian J Med Biol Res. 2012;45(7):591–600. DOI: 10.1590/s0100-879x2012007500059 PMC3854278 22510779

[20] Sherk VD, Bemben MG, Bemben DA. Interlimb muscle and fat comparisons in persons with lower-limb amputation. Arch Phys Med Rehabil. 2010;91(7):1077–81. DOI: 10.1016/j.apmr.2010.04.008 20599046

[21] Okorodudu DO, Jumean MF, Montori VM, Romero-Corral A, Somers VK, Erwin PJ, et al. Diagnostic performance of body mass index to identify obesity as defined by body adiposity: a systematic review and meta-analysis. Int J Obes. 2010;34(5):791–9. DOI: 10.1038/ijo.2010.5 20125098

[22] Ward LC. Bioelectrical impedance analysis for body composition assessment: reflections on accuracy, clinical utility, and standardisation. Eur J Clin Nutr. 2019;73(2):194–9. DOI: 10.1038/s41430-018-0335-3 30297760

[23] Flakoll P, Kent P, Neyra R, Levenhagen D, Chen K, Ikizler T. Bioelectrical impedance vs air displacement plethysmography and dual-energy X-ray absorptiometry to determine body composition in patients with end-stage renal disease. J Parenter Enter Nutr. 2004;28(1):13–21. DOI: 10.1177/014860710402800113 14763788

[24] Lopez-Gonzalez D, Wells JCK, Clark P. Body composition assessment in Mexican children and adolescents. part 2: cross-validation of three bio-electrical impedance methods against dual X-ray absorptiometry for total-body and regional body composition. Nutrients. 2022;14(5):965. DOI: 10.3390/nu14050965 35267947 PMC8912617

[25] Gomez-Arbelaez D, Bellido D, Castro AI, Ordoñez-Mayan L, Carreira J, Galban C, et al. Body Composition changes after very-low-calorie ketogenic diet in obesity evaluated by 3 standardized methods. J Clin Endocrinol Metab. 2017;102(2):488–98. DOI: 10.1210/jc.2016-2385 27754807

[26] Ziai S, Coriati A, Chabot K, Mailhot M, Richter MV, Rabasa-Lhoret R. Agreement of bioelectric impedance analysis and dual-energy X-ray absorptiometry for body composition evaluation in adults with cystic fibrosis. J Cyst Fibros. 2014;13(5):585–8. DOI: 10.1016/j.jcf.2014.01.006 24522087

[27] Kyle U, Genton L, Hans D, Karsegard L, Slosman D, Pichard C. Age-related differences in fat-free mass, skeletal muscle, body cell mass and fat mass between 18 and 94 years. Eur J Clin Nutr. 2001;55(8):663–72. DOI: 10.1038/sj.ejcn.1601198 11477465

[28] Lowry DW, Tomiyama AJ. Air displacement plethysmography versus dual-energy x-ray absorptiometry in underweight, normal-weight, and overweight/obese individuals. PLoS One. 2015;10(1):e0115086. DOI: 10.1371/journal.pone.0115086 25607661 PMC4301864

[29] Morgan SJ, Balkman GS, Gaunaurd IA, Kristal A, Amtmann D, Hafner BJ. Clinical resources for assessing mobility of people with lower-limb amputation: interviews with rehabilitation clinicians. J Prosthet Orthot. 2022;34(2):69–78. DOI: 10.1097/jpo.0000000000000345 35431518 PMC9007274

[30] Maggioni M, Bertoli S, Margonato V, Merati G, Veicsteinas A, Testolin G. Body composition assessment in spinal cord injury subjects. Acta Diabetol. 2003;40:s183–6. DOI: 10.1007/s00592-003-0061-7 14618468

[31] Beck LA, Lamb JL, Atkinson EJ, Wuermser L-A, Amin S. Body composition of women and men with complete motor paraplegia. J Spinal Cord Med. 2014;37(4):359–65. DOI: 10.1179/2045772313Y.0000000151 24090208 PMC4116716

[32] Spungen AM, Wang J, Pierson RN, Bauman WA. Soft tissue body composition differences in monozygotic twins discordant for spinal cord injury. J Appl Physiol. 2000;88(4):1310–5. DOI: 10.1152/jappl.2000.88.4.1310 10749824

[33] Swisher A, Yeater R, Moffett K, Baer L, Stanton B. A comparison of methods to determine body fat in individuals with cystic fibrosis: a pilot study. J Exerc Physiol Online. 2003;6(2).

[34] Siri WE. Body composition from fluid spaces and density: analysis of methods. 1961. Nutrition. 1993 Sep-Oct;9(5):480–91; discussion 480, 492. PMID: 8286893 8286893

[35] Brozek J, Grande F, Anderson JT, Keys A. Densitometric analysis of body composition: revision of some quantitative assumptions. Ann N Y Acad Sci. 1963;110:113–40. DOI: 10.1111/j.1749-6632.1963.tb17079.x 14062375

[36] van Beijsterveldt IALP, Beunders VAA, Bijlsma A, Vermeulen MJ, Joosten KFM, Hokken-Koelega ACS. Body composition assessment by air-displacement plethysmography compared to dual-energy X-ray absorptiometry in full-term and preterm aged three to five years. J Clin Med. 2022;11(6):1604. DOI: 10.3390/jcm11061604 35329930 PMC8952802

[37] Delisle-Houde P, Reid RER, Insogna JA, Prokop NW, Buchan TA, Fontaine SL, et al. Comparing DXA and air displacement plethysmography to assess body composition of male collegiate hockey players. J Strength Cond Res. 2019;33(2):474–8. DOI: 10.1519/JSC.0000000000001863 28234718

[38] Lowry DW, Tomiyama AJ. Air Displacement Plethysmography versus Dual-Energy X-Ray Absorptiometry in Underweight, Normal-Weight, and Overweight/Obese Individuals. Thearle M, editor. PLoS One. 2015;10(1):e0115086. DOI: 10.1371/journal.pone.0115086 PMC4301864 25607661

[39] Ballard TP, Fafara L, Vukovich MD. Comparison of Bod Pod and DXA in female collegiate athletes. Med Sci Sports Exerc. 2004;36(4):731–5. DOI: 10.1249/01.mss.0000121943.02489.2b 15064602

[40] Collins MA, Millard-Stafford ML, Sparling PB, Snow TK, Rosskopf LB, Webb SA, et al. Evaluation of the BOD POD for assessing body fat in collegiate football players. Med Sci Sports Exerc. 1999;31(9):1350–6. DOI: 10.1097/00005768-199909000-00019 10487380

[41] Wechsler, S. Statistics at Square One. 9th ed, revised by M. J. Campbell, T. D. V. Swinscow, BMJ Publ. Group, London, ISBN 0-7279-0916-9. Statistics in Medicine. 1996; 16(22): 2629–2630

[42] Vincent W. Statistics in Kinesiology - 3rd ed. Champaign: Human Kinetics; 2004.

[43] Cohen, J. Statistical Power Analysis for the Behavioral Sciences - 2nd ed. Routledge. 1988; DOI: 10.4324/9780203771587

[44] Fields DA, Goran MI, McCrory MA. Body-composition assessment via air-displacement plethysmography in adults and children: a review. Am J Clin Nutr. 2002;75(3):453–67. DOI: 10.1093/ajcn/75.3.453 11864850

[45] Miyatake N, Nonaka K, Fujii M. A new air displacement plethysmograph for the determination of Japanese body composition. Diabetes, Obes Metab. 1999;1(6):347–51. DOI: 10.1046/j.1463-1326.1999.00064.x 11225651

[46] Ritz P, Sallé A, Audran M, Rohmer V. Comparison of different methods to assess body composition of weight loss in obese and diabetic patients. Diabetes Res Clin Pract. 2007;77(3):405–11. DOI: 10.1016/j.diabres.2007.01.007 17306903

[47] Goosey-Tolfrey V, Keil M, Brooke-Wavell K, de Groot S. A Comparison of methods for the estimation of body composition in highly trained wheelchair games players. Int J Sports Med. 2016;37(10):799–806. DOI: 10.1055/s-0042-104061 27176890

[48] Gibson AL, Heyward VH, Mermier CM, Janot JM, Wilmerding MV. Comparison of DXA, Siri's 2C, and Lohman's Db-Mineral models for estimating the body fat of physically active adults. Int J Sport Nutr Exerc Metab. 2004;14(6):657–72. DOI: 10.1123/ijsnem.14.6.657 15657471

[49] Deurenberg P, Leenen R, Van der Kooy K, Hautvast JG. In obese subjects the body fat percentage calculated with Siri's formula is an overestimation. Eur J Clin Nutr. 1989;43(8):569–75. 2598896

[50] Vescovi JD, Zimmerman SL, Miller WC, Fernhall B. Effects of clothing on accuracy and reliability of air displacement plethysmography. Med Sci Sports Exerc. 2002;34(2):282–5. DOI: 10.1097/00005768-200202000-00016 11828238

[51] Hull HR, Fields DA. Effect of short schemes on body composition measurements using Air-Displacement Plethysmography. Dyn Med. 2005;4(1):8. DOI: 10.1186/1476-5918-4-8 16045792 PMC1187904

[52] Frisard MI, Greenway FL, DeLany JP. Comparison of methods to assess body composition changes during a period of weight loss. Obes Res. 2005;13(5):845–54. DOI: 10.1038/oby.2005.97 15919837

[53] Weyers AM, Mazzetti SA, Love DM, Gómez Anal, Kraemer WJ, Volek JS. Comparison of methods for assessing body composition changes during weight loss. Med Sci Sport Exerc. 2002;34(3). DOI: 10.1097/00005768-200203000-00017 11880815

[54] Minderico CS, Silva AM, Teixeira PJ, Sardinha LB, Hull HR, Fields DA. Validity of air-displacement plethysmography in the assessment of body composition changes in a 16-month weight loss program. Nutr Metab (Lond) 2006;3(1):32. DOI: 10.1186/1743-7075-3-32 16925811 PMC1560140

[55] Pietrobelli A, Wang Z, Formica C, Heymsfield SB. Dual-energy X-ray absorptiometry: fat estimation errors due to variation in soft tissue hydration. Am J Physiol. 1998;274(5):E808–16. DOI: 10.1152/ajpendo.1998.274.5.E808 9612238

[56] Pietrobelli A, Formica C, Wang Z, Heymsfield SB. Dual-energy X-ray absorptiometry body composition model: review of physical concepts. Am J Physiol Metab. 1996;271(6):E941–51. DOI: 10.1152/ajpendo.1996.271.6.E941 8997211

[57] Al Yafi M, Nasif A, Glosser LD, Ren G, Ahemd A, Nazzal M, et al. The relationship between lower extremity amputation and body mass index. Vascular. 2022;170853812210878. DOI: 10.1177/17085381221087824 35451901

